# Aspartame carcinogenic potential revealed through network toxicology and molecular docking insights

**DOI:** 10.1038/s41598-024-62461-w

**Published:** 2024-05-20

**Authors:** Dandan Chen, Xianbing Hou

**Affiliations:** Fenghua Hospital of Traditional Chinese Medicine, Ningbo, Zhejiang China

**Keywords:** Network toxicology, Molecular docking, Sweetener, Aspartame, Carcinogenicity, Mechanism of action, Chemical safety, Biomarkers, Gastroenterology, Oncology

## Abstract

The research employed network toxicology and molecular docking techniques to systematically examine the potential carcinogenic effects and mechanisms of aspartame (l-α-aspartyl-l-phenylalanine methyl ester). Aspartame, a commonly used synthetic sweetener, is widely applied in foods and beverages globally. In recent years, its safety issues, particularly the potential carcinogenic risk, have garnered widespread attention. The study first constructed an interaction network map of aspartame with gastric cancer targets using network toxicology methods and identified key targets and pathways. Preliminary validation was conducted through microarray data analysis and survival analysis, and molecular docking techniques were employed to further examine the binding affinity and modes of action of aspartame with key proteins. The findings suggest that aspartame has the potential to impact various cancer-related proteins, potentially raising the likelihood of cellular carcinogenesis by interfering with biomolecular function. Furthermore, the study found that the action patterns and pathways of aspartame-related targets are like the mechanisms of known carcinogenic pathways, further supporting the scientific hypothesis of its potential carcinogenicity. However, given the complexity of the in vivo environment, we also emphasize the necessity of validating these molecular-level findings in actual biological systems. The study introduces a fresh scientific method for evaluating the safety of food enhancers and provides a theoretical foundation for shaping public health regulations.

## Introduction

Aspartame, also called l-α-aspartyl-l-phenylalanine methyl ester, is an artificial sweetener with few calories. Since its discovery in 1965, it has been approved in over ninety countries worldwide for use in thousands of food products and beverages. These products include sugar-free sodas, diet drinks, low-sugar or sugar-free desserts, chewing gum, flavor packets, certain medications, and energy drinks^1^. Due to its sweetness being around two hundred times greater than sucrose, only a small quantity is needed to reach the desired level of sweetness in food items, which is why it is favored by diabetics and individuals aiming to shed pounds^2^. However, there remains controversy among the public and the scientific community regarding the health risks associated with long-term consumption, particularly its carcinogenicity. This controversy persists even after the World Health Organization (WHO) classified aspartame as a carcinogenic substance^3^. The U.S. Food and Drug Administration (FDA) continues to regard aspartame as safe within the recommended intake limits, setting the Acceptable Daily Intake (ADI) at 40 mg per kilogram of body weight^4^. Part of the reason for this controversy is that early toxicological studies were based on high-dose animal experiments and could not be directly inferred to low-dose human exposure^5^. Additionally, inconsistencies in the results of different studies, as well as limited understanding of aspartame's metabolic pathways and the mechanisms of action of its metabolites, add to the complexity of assessing its safety^6^. Furthermore, additional research indicates that aspartame may pose a risk factor for diseases such as cerebrovascular disorders^7^, depression^8^, and autism^9^. Currently, there is still controversy regarding the safety of aspartame in epidemiological research findings, with some studies suggesting it as a risk factor for tumors while others reach the opposite conclusion^10,11^. With the advancement of toxicological research methods, especially the emergence of network toxicology, researchers have begun to analyze from a systems biology perspective how chemicals affect the stability of biological systems. Network toxicology integrates bioinformatics, systems biology, and toxicology to systematically study the interactions between chemicals and biological systems at the molecular level, and how these interactions lead to toxic effects^12^. In this study, we combine network toxicology with molecular docking strategies to investigate the potential carcinogenicity of aspartame and its molecular mechanisms of action, with preliminary validation through microarray data analysis and survival analysis. We hope that this multidisciplinary approach will provide a more in-depth and comprehensive scientific basis for the safety assessment of aspartame and offer a new research paradigm for the safety studies of other sweeteners and food additives.

## Material and methods

### Targets for aspartame collection

The standard structure and Canonical SMILES of aspartame were identified by searching for "aspartame" in the PubChem database. Using the search results, potential targets of aspartame were anticipated by utilizing databases like ChEMBL 33^13^, STITCH (Version 5.0)^14^, Swiss Target Prediction^15^, Similarity ensemble approach^16^, and PharmMapper (Version 2017)^17^, focusing on the keyword 'aspartame' and limited to the species 'Homo sapiens'. We examined the structure of the search results and compared their consistency. Integrate and remove duplicate data of the potential targets found in the search, including ChEMBL numbers and STITCH codes. Standardize the obtained target names using the Uniprot database^18^. We searched these databases up to February 5, 2024.

### Collection of gastric cancer targets

Targets related to gastric cancer were gathered by searching the GeneCard (Version 5.19)^19^, OMIM^20^, and DisGeNET (v7.0) databases^21^ using the keyword 'gastric cancer'. Furthermore, we used Venn diagrams to filter the common potential targets between aspartame and gastric cancer, considering the intersecting parts as potential targets for aspartame. We searched these databases up to February 5, 2024.

### Screening of core targets and construction of protein interaction networks

Data on potential links between aspartame consumption and gastric cancer were entered into the STRING (Version: 12.0) database^22^. Only 'Homo sapiens' were included in the species, with a 'medium confidence (0.400)' set as the minimum required interaction score, and any disconnected nodes in the network were not displayed. Subsequently, the target genes were analyzed. Information produced by STRING was brought into Cytoscape (v3.10.1)^23,24^ to construct a protein–protein interaction (PPI) network chart, with the core targets chosen according to their degree values utilizing the cytoHubba plugin.

### Functional analysis of target proteins and pathway enrichment

To examine the biological roles of the possible targets for gastric cancer triggered by aspartame, information was gathered from the DAVID (v2023q4) database^25^ for analysis of Gene Ontology (GO) and enrichment analysis of Kyoto Encyclopedia of Genes and Genomes (KEGG) pathways. The analysis of gene ontology (GO) included evaluations of BP, CC, and MF to reveal the main biological functions of the associated targets. Significant pathways related to potential gastric cancer targets induced by aspartame were identified through KEGG enrichment analysis^26^. A significance level of corrected p-value < 0.05 was used to determine the primary pathways of the identified targets, with data visualization done using the SRplot platform^27^.

### Microarray data analysis

In order to confirm the findings of this research, we obtained two microarray datasets, GSE54129^28^ and GSE63089^29^, from the Gene Expression Omnibus-NCBI (GEO) database^30^. GEO serves as a public database for gene expression data obtained through high-throughput methods, including chips, microarrays, and hybridization arrays. Afterward, by utilizing GEO2R for examination, we acquired distinct gene expression information comparing gastric cancer with healthy tissues. The SRplot platform was utilized to create volcano plots for visualizing the genes that were expressed differentially, with criteria for selection being corrected p-value < 0.05 and |log (FC)|  ≥ 1. Following this, the differentially expressed genes (DEGs) were compared with hub genes, and intersecting genes were selected for further analysis.

### Survival analysis

The Kaplan–Meier Plotter^31^ was utilized to perform survival analysis on the last crucial hub genes. Based on gene expression levels, survival analysis can also assess the clinical significance of a specific gene. Kaplan–Meier survival curves were created in this database to analyze the correlation between hub genes (MMP9, CASP3) and the survival rate of patients with gastric cancer.

### Molecular docking analysis

**Figure 1:**
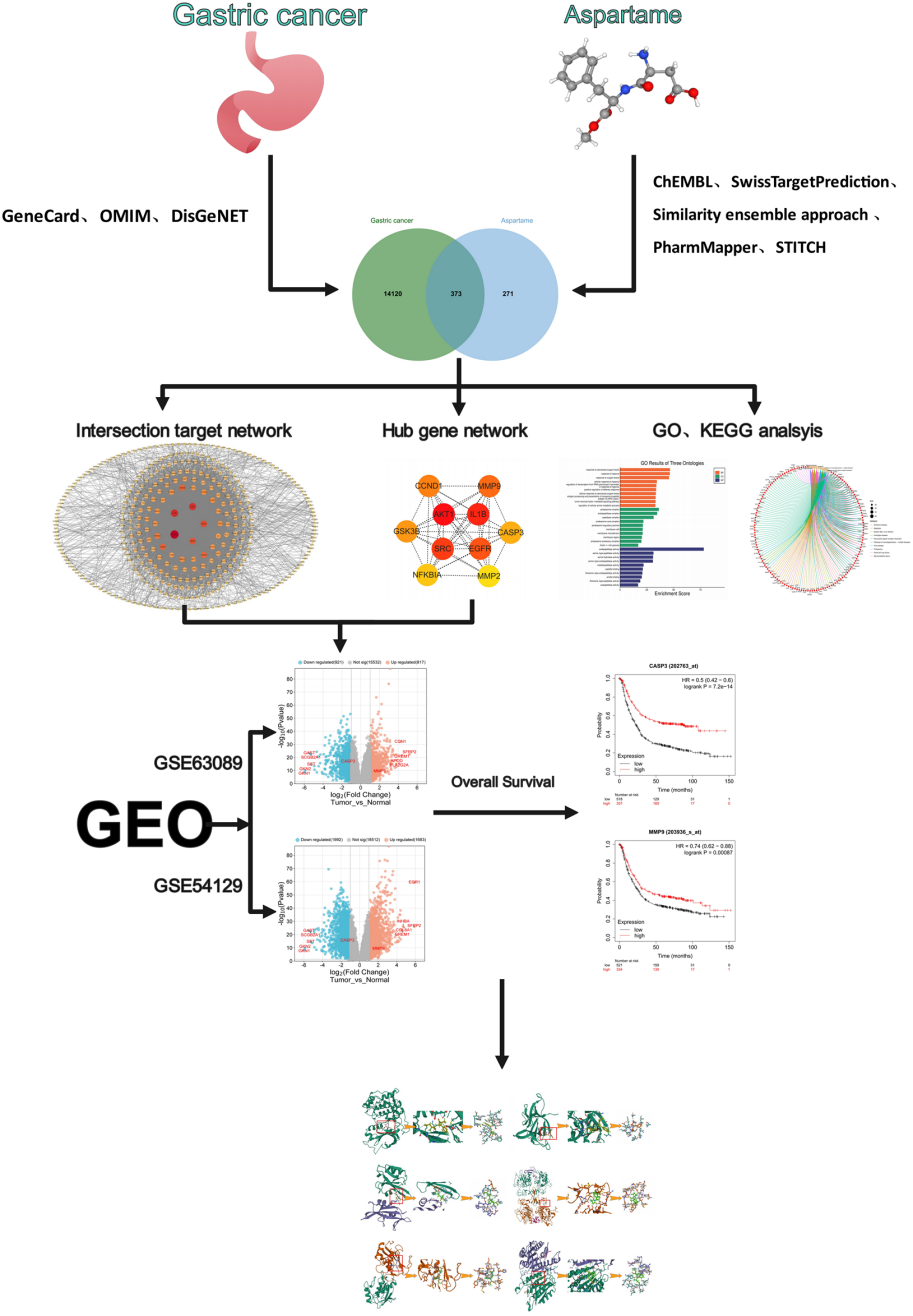
Flow chart of the study.

To assess the binding affinity and interaction patterns between aspartame and its targets, we utilized Autodock Vina 1.2.2^32^.The molecular structure of aspartame was obtained from the PubChem compound database^33^.Protein AKT1, IL1B, SRC, EGFR, MMP9, and CASP3 were obtained from the Protein Data Bank (PDB)^34^ and their 3D coordinates were downloaded. Initially, we converted all protein and ligand files to PDBQT format, eliminating water molecules and introducing polar hydrogen atoms. The grid box was positioned at the center to encompass the structural regions of every protein and allow for unrestricted molecular motion. The docking pocket was configured as a 30 Å × 30 Å × 30 Å cubic space, with grid points spaced 0.05 nm apart. Autodock Vina 1.2.2 (http://autodock.scripps.edu/) was utilized to visualize the molecular docking investigations. The flow chart for the study is displayed in Fig. 1 below.

## Result

### Determination of potential targets for aspartame and gastric cancer

**Figure 2:**
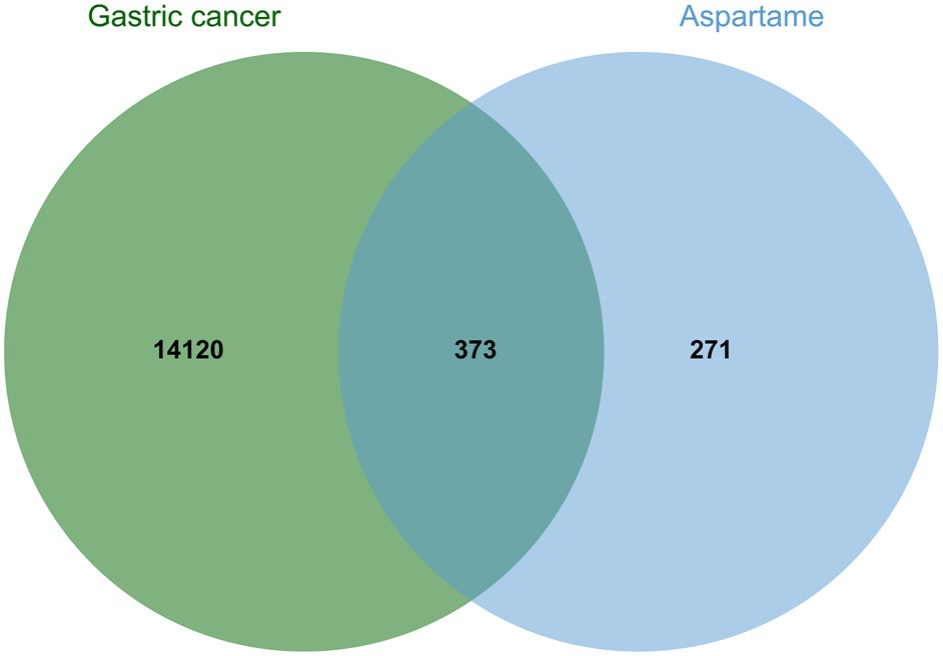
Venn diagram of the targets of Aspartame and Gastric cancer.

A preliminary selection of 644 aspartame-acting targets was made from CHEMBL, STTCH, Swiss Target Prediction, Similarity ensemble approach and PharmMapper, and 14,493 targets associated with gastric cancer were identified through the GeneCard, OMIM, and DisGeNET databases. By integrating these target collections and removing duplicate data, a total of 373 intersecting targets were obtained, serving as potential targets consistent with the carcinogenicity of aspartame in gastric cancer (Fig. 2).

### Acquisition of core targets and interaction network with potential targets

**Figure 3:**
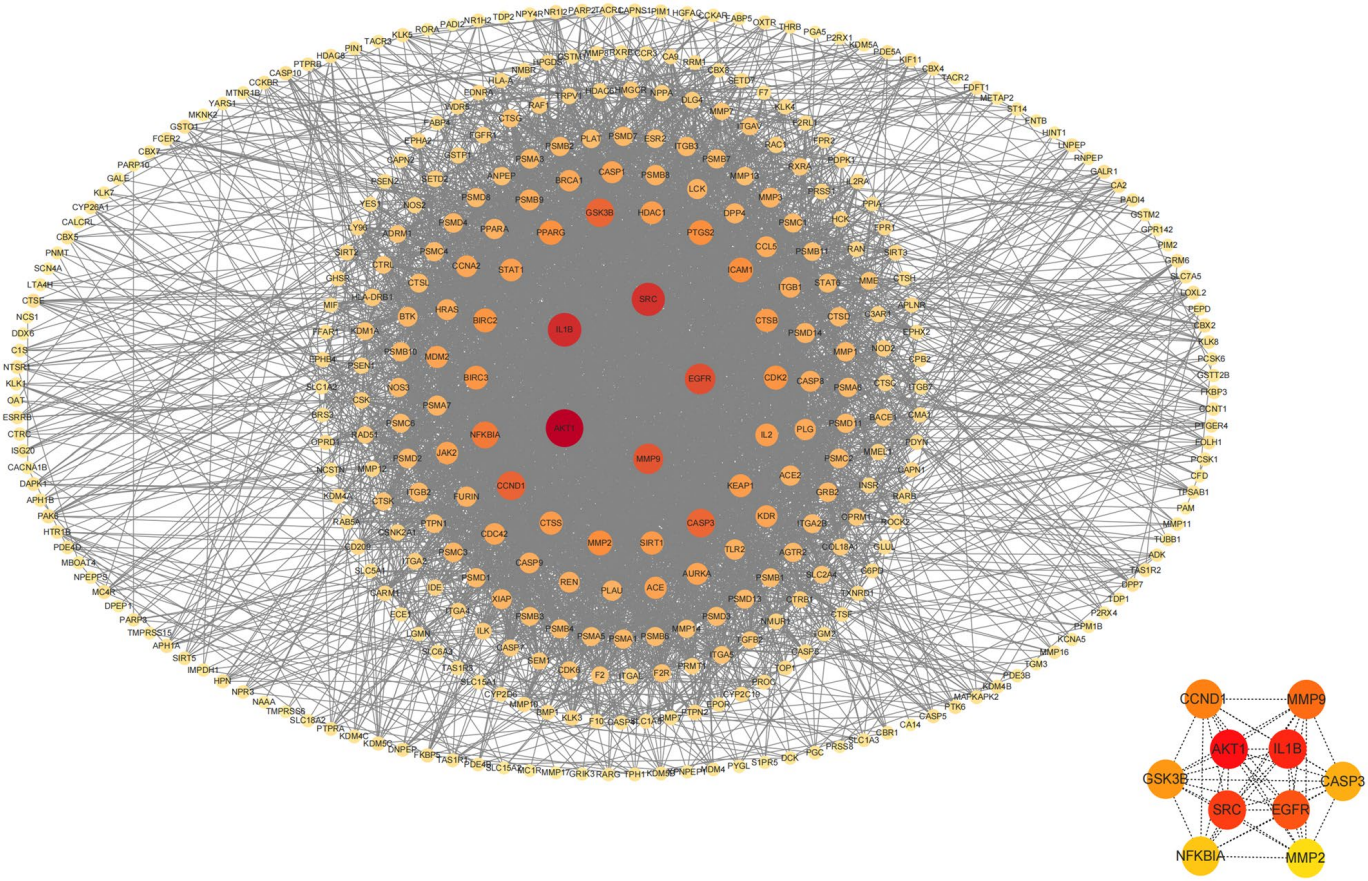
The PPI network of the potential targets and the core targets.

A network of protein interactions (PPI) was built by utilizing the STRING database with a confidence level of 0.4, leading to a network containing 366 nodes and 4294 edges, and an average degree of 23.464. Simultaneously, Cytoscape software was utilized to create a visualization of a protein–protein interaction network map (Fig. 3). A map of protein–protein interactions(Fig. 3) was created to demonstrate the specific connections among the 10 main targets identified through network analysis in gastric cancer (Table 1).

**Table 1:** Core targets selected from the PPI network.

Gene	Degree	Average shortest PathLength	Betweenness centrality	Closeness centrality	Neighborhood connectivity	Topological coefficient
AKT1	151	1.610958904	0.10887566	0.620748299	35.74834437	0.100416698
IL1B	127	1.693150685	0.070541442	0.590614887	37.8976378	0.108278965
SRC	124	1.715068493	0.095376995	0.583067093	35.54032258	0.103015428
EGFR	109	1.736986301	0.047344548	0.575709779	40.11926606	0.113975188
MMP9	105	1.78630137	0.031027472	0.559815951	42.39047619	0.125045652
CCND1	98	1.832876712	0.033583765	0.545590433	46.43877551	0.140723562
GSK3B	97	1.838356164	0.037787154	0.543964232	45.90721649	0.140788059
CASP3	96	1.794520548	0.03057402	0.557251908	44.17708333	0.128421754
NFKBIA	86	1.871232877	0.020673557	0.534407028	49.23255814	0.150558282
MMP2	75	1.890410959	0.01224751	0.528985507	48.34666667	0.146062437

### Target functional analysis and pathway enrichment analysis

**Figure 4:**
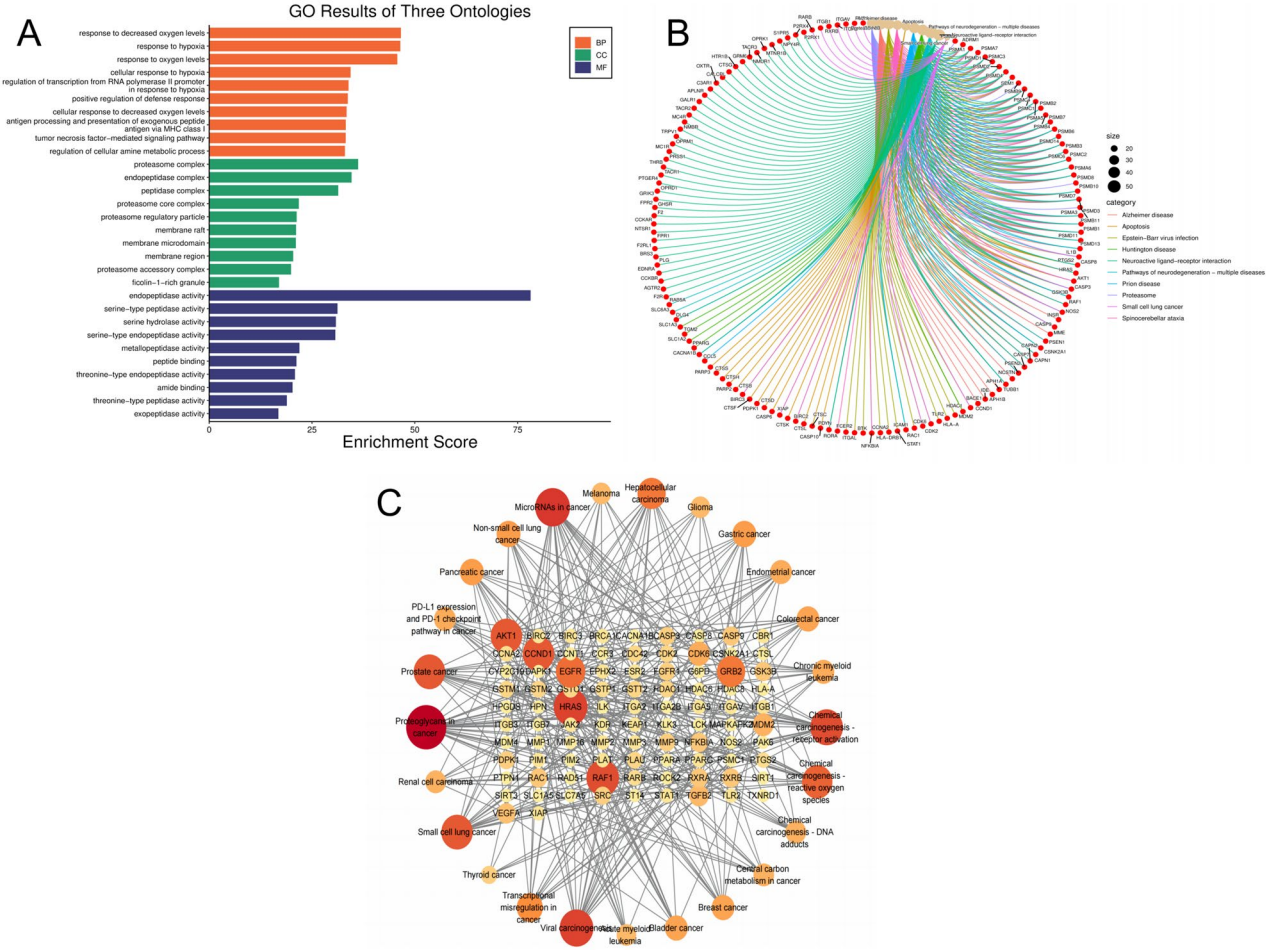
Enrichment analysis diagram. (**A**) GO Enrichment analysis of potential targets (top 10); (**B**) KEGG enrichment analysis of potential targets (top 10); (**C**) network map of tumor-related pathways and targets.

Analysis using Gene Ontology (GO) and Kyoto Encyclopedia of Genes and Genomes (KEGG) was performed on 366 potential targets, focusing specifically on Homo sapiens. The analysis yielded 2609 statistically significant GO terms, encompassing 2221 biological processes (BP), 146 cellular components (CC), and 242 molecular functions (MF).Ranking of GO terms was determined by their Score values, with the top 10 terms in Biological Process (BP), Cellular Component (CC), and Molecular Function (MF) chosen for visualization (Fig. 4A).At the same time, an analysis using KEGG was conducted to identify their participation in particular signaling pathways. Out of 142 pathways, the top 10, sorted by p-value, were visualized (Fig. 4B). Of the 142 pathways, 25 were cancer-related, with certain core genes implicated in the pathogenesis and progression of cancer (Fig. 4C).

### Microarray data analysis

**Figure 5:**
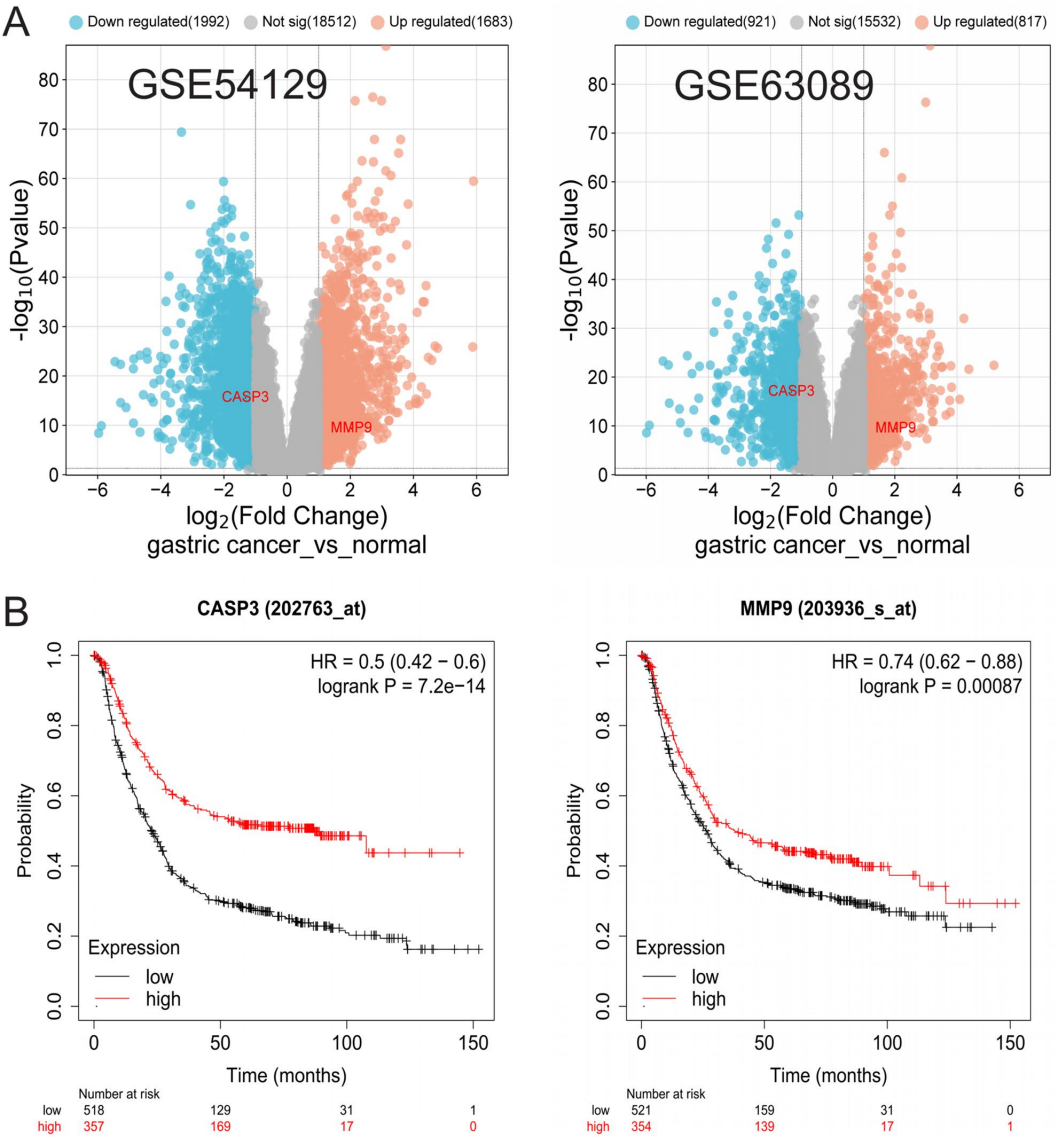
Related data set differential gene analysis. (**A**) Volcano plot of the differential genes; (**B**) survival analysis diagram.

From the GEO database, 22,187 differential genes were obtained from the GSE54129 dataset, and 17,270 differential genes from the GSE63089 dataset. Intersection with ten hub genes yielded two intersecting genes, with MMP9 being upregulated and CASP3 downregulated. The differential genes from the GEO datasets were visualized using the Microsynth platform, as shown in Fig. 5A.

### Survival analysis

The Kaplan–Meier method was employed to estimate survival functions. After constructing Kaplan–Meier survival curves, the log-rank test was used to compare the survival rates of the high-expression and low-expression groups. The analysis suggests that the MMP9 and CASP3 genes may influence the overall survival (OS) in gastric cancer (Fig. 5B).

### Molecular docking of aspartame to the core targets

**Figure 6:**
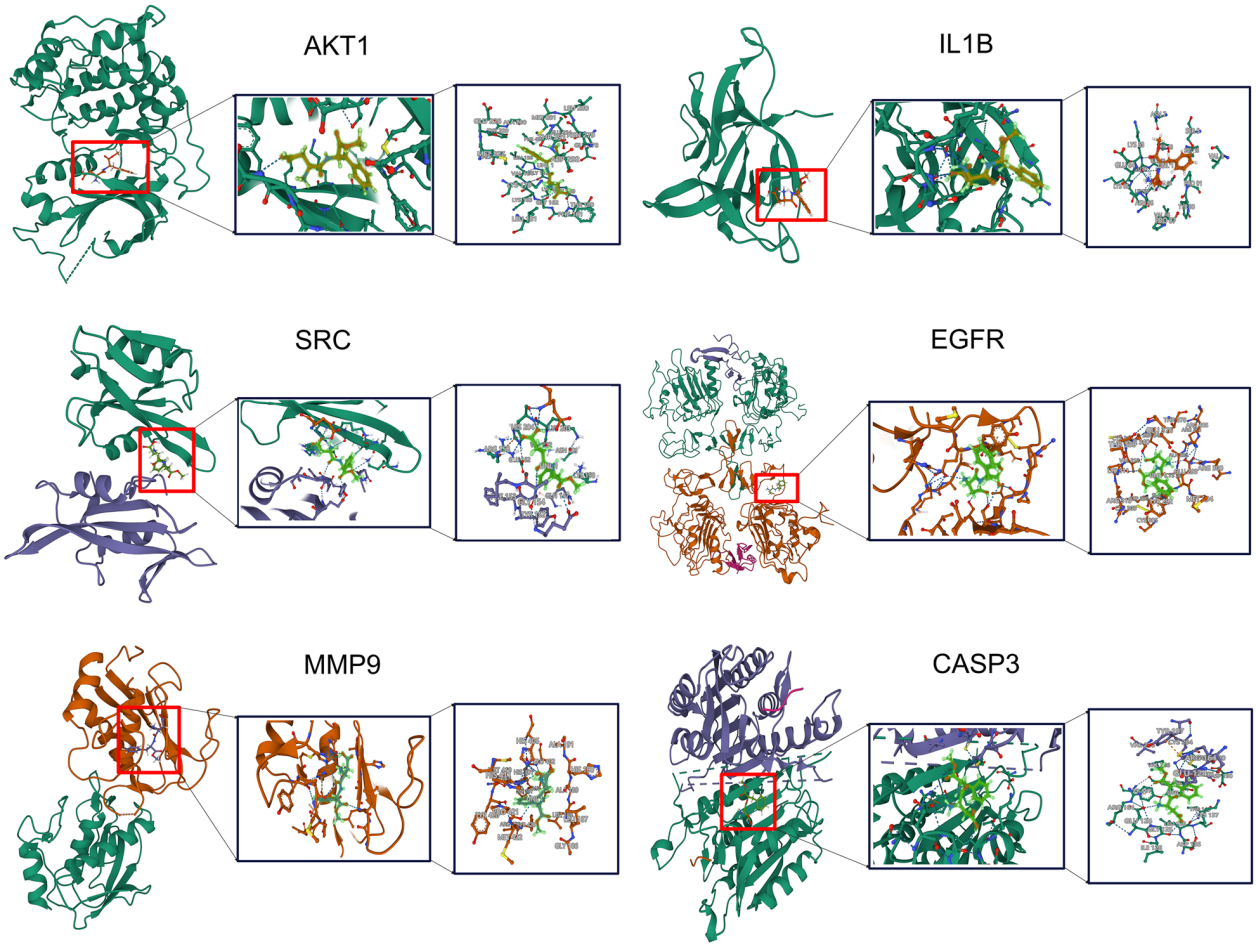
Molecular docking results with the lowest aspartame binding energy for each target.

To evaluate the affinity of aspartame with its targets, molecular docking analysis was performed. The binding poses and interactions of aspartame with six proteins were obtained using Autodock Vina version 1.2.2, resulting in the binding energy for each interaction being generated (see Fig. 6 and Table 2). The findings suggest that aspartame forms noticeable hydrogen bonds and robust electrostatic interactions with its protein targets. For aspartame, the low binding energies observed with all six proteins suggest a highly stable binding.

**Table 2:** Binding energy for target with aspartame.

Target	Degree	PDB number	Hydrone	Binding energy (kcal/mol)
AKT1	151	4EKL	Aspartame	− 6.521
IL1B	127	1HIB	Aspartame	− 5.538
SRC	124	1A07	Aspartame	− 6.821
EGFR	109	1IVO	Aspartame	− 6.511
MMP9	105	1GKC	Aspartame	− 7.75
CASP3	96	1CP3	Aspartame	− 6.365

## Discussion

The study presents convincing proof indicating a link between the artificial sweetener aspartame and the occurrence of stomach cancer. Through the use of a comprehensive strategy that merges molecular biology methods with bioinformatics analysis, we have discovered possible connections between aspartame and particular cancer indicators, evaluating the expression profiles of these indicators in gastric cancer and their impact on patient outcomes.

Aspartame may promote the progression of gastric cancer by affecting multiple keys signaling proteins and regulatory factors. Key proteins like AKT1, IL1B, SRC, EGFR, MMP9, CCND1, GSK3B, CASP3, NFKBIA, and MMP2 play roles in various biological functions such as cell growth, cell death, immune response, cell attachment, and restructuring of the extracellular matrix.

AKT1 is an essential element in the PI3K/AKT/mTOR signaling pathway, crucial for controlling cell survival and death, cellular growth, metabolism, and the development and metastasis of tumors. The activation of this pathway is linked to unfavorable clinical outcomes in various cancer types, such as gastric cancer^35^. IL1B: The gene's protein product is a cytokine involved in the body's response to inflammation. Inflammation has been established as a significant driving factor in the development of cancer, particularly in cancers associated with chronic inflammation, such as gastric cancer^36^. SRC: As a non-receptor tyrosine kinase, SRC plays a role in cellular proliferation, differentiation, migration, and survival. In cancer cells, SRC is often aberrantly activated, promoting tumor growth and metastasis^37^. Overexpression of EGFR, the Epidermal Growth Factor Receptor, has been detected in distinct types of cancers, such as gastric cancer, and is linked to the growth and spread of tumors^38^. Overexpression of CCND1 can result in uncontrolled cell proliferation as it plays a crucial role in regulating the cell cycle^39^. GSK3B plays a role in controlling different cellular processes, such as cell division and programmed cell death. Dysregulation of GSK3B in cancer may lead to uncontrolled cell growth^40^. NFKBIA suppresses the NF-κB pathway, which is crucial for controlling inflammation, immune response, and cell viability^41^. NF-κB activation is linked to the growth and advancement of different types of cancer. Just like MMP9, MMP2 plays a role in breaking down the extracellular matrix, which helps tumors invade and spread^42^.

According to the analysis of potential targets by GO and KEGG, these genes exhibit a broad distribution and expression in various subcellular localizations and play a significant role in cellular responses to changes in oxygen levels, particularly under hypoxic stress and anoxia (as in certain types of cancer).The proteins encoded by these genes are primarily associated with the proteasome, involved in protein degradation, antigen processing and presentation, and signal transduction. They might also have a significant impact on different neurodegenerative conditions, possibly because of the strong connection between proteasome activity, protein clumping, and cell death. Furthermore, these genes may influence tumor progression and immune system function through their involvement in immune responses and inflammatory signaling pathways. This information is crucial for understanding the molecular mechanisms of diseases and potential therapeutic targets (Fig. S1)^43^.

We noticed an increase in MMP9 and a decrease in CASP3 when comparing the gene expression patterns of healthy stomach tissue with those of stomach cancer tissue. This may suggest that in gastric cancer, there is an enhanced degradation of the extracellular matrix and an increased invasive capability of the tumor (indicated by the upregulation of MMP9), while the mechanisms of cell apoptosis are simultaneously inhibited (indicated by the downregulation of CASP3). The expression levels of MMP9 and CASP3 are significantly correlated with the overall survival time of patients with gastric cancer. Specifically, high expression of MMP9 may be associated with a poorer prognosis, while low expression of CASP3 may further exacerbate the malignancy of the tumor.

Matrix metalloproteinase 9 (MMP9) is a key enzyme involved in degrading the extracellular matrix (ECM), responsible for breaking down various components within the ECM, including collagen and fibronectin. During the development of gastric cancer, the increased activity of MMP9 is crucial for tumor cells to penetrate the basement membrane and ECM, facilitating tumor cell invasion into neighboring tissues and their dissemination to new sites through the bloodstream or lymphatic system^44^. By degrading ECM, MMP9 not only alters the physical and chemical environment surrounding tumor cells, thereby promoting tumor cell proliferation and survival, but also influences the tumor immune microenvironment. It indirectly regulates the infiltration and function of immune cells by adjusting the composition and structure of ECM, thereby impacting the overall immune response^45^. Furthermore, through its degradative action on ECM, MMP9 paves the way for the formation of new blood vessels within tumors, which is crucial for tumor blood supply, oxygen delivery, as well as further growth and dissemination^46^. The expression and activity of MMP9 are regulated by various tumor-related factors, including TGF-β, EGF, and FGF, which participate in the regulation of multiple signaling pathways involved in tumor initiation and progression^47^. In some cases, MMP9 is also associated with the resistance of tumor cells to chemotherapy drugs, possibly by altering the composition and density of the ECM, thereby affecting the distribution and efficacy of drugs^48^. It is worth noting that MMP9 does not act in isolation; there is a complex mutual regulatory relationship between MMP9 and other members of the MMP family and their inhibitors (TIMPs).

Caspase-3 (CASP3) plays a core role in the process of cell apoptosis, which is a precisely regulated mechanism of cellular self-destruction crucial for maintaining tissue health and physiological balance. In the progression of cancer, especially in gastric cancer development, the apoptosis pathway is often disrupted, a common feature of various malignant tumors^49^. Inhibition of CASP3 activity may lead to increased resistance of gastric cancer cells to certain chemotherapy drugs, thereby reducing the effectiveness of treatment^50^. Moreover, the complex interaction between tumor cells and their microenvironment, including surrounding non-tumor cells, extracellular matrix, and signaling molecules, may be affected by abnormal CASP3 function. This abnormality may not only disrupt the invasiveness and metastatic potential of tumors but also impact the overall growth dynamics of tumors. Although closely associated with cell death, CASP3 has also been shown to promote the migration and invasion of tumor cells through non-traditional pathways other than apoptosis under specific conditions^51,52^. Additionally, in certain circumstances, the expression level of CASP3 is closely related to the prognosis of gastric cancer patients, with reduced expression often associated with higher tumor grades, poor prognosis, and lower survival rates in patients^53^.

We also conducted molecular docking studies, a computational method for simulating the binding of drug molecules to their target proteins. A decrease in docking energy indicates a higher level of interaction between the molecules, resulting in a more secure binding. Our results indicate that the binding energies of aspartame to these key proteins range from − 5.538 to − 7.75 kcal/mol, with the highest binding energy to MMP9 (− 7.75 kcal/mol), suggesting a potentially strong interaction between aspartame and MMP9.This is consistent with the findings of the upregulated expression of the MMP9 gene.

## Conclusions

The findings suggest that aspartame has the potential to impact various cancer-related proteins, potentially raising the likelihood of cellular carcinogenesis by interfering with biomolecular function. Furthermore, the study found that the action patterns and pathways of aspartame-related targets are like the mechanisms of known carcinogenic pathways, further supporting the scientific hypothesis of its potential carcinogenicity.

In summary, our study underscores the importance of continued investigation into the relationship between artificial sweeteners and cancer, which may impact the diagnostic and therapeutic strategies for gastric cancer. Future research should focus on revealing how aspartame contributes to the development of gastric cancer by affecting cellular signaling pathways, as well as how this knowledge can be utilized to develop novel treatment modalities.

While our research offers fresh perspectives on the possible connection between aspartame and stomach cancer, there are constraints to consider. Firstly, our research is based on observational biomarker expression patterns, which limits our interpretation of causality. Secondly, while microarray data is a powerful tool, its results need to be validated through independent methods such as quantitative PCR, Western Blotting, or immunohistochemistry. Additionally, our results need to be confirmed in larger, separate groups and supported by epidemiological research to determine the connection between consuming aspartame and the likelihood of developing gastric cancer.

### Supplementary Information

Supplementary Figure S1.

Supplementary Legends.

## Data Availability

All data supporting the findings of this study are available within the paper and its Supplementary Information.
