# Supplementary figures and images for: Aspartame carcinogenic potential revealed through network toxicology and molecular docking insights
Source: Sci Rep. 2024 May 20;14:11492. doi: 10.1038/s41598-024-62461-w (PMC11106323; doi:10.1038/s41598-024-62461-w)

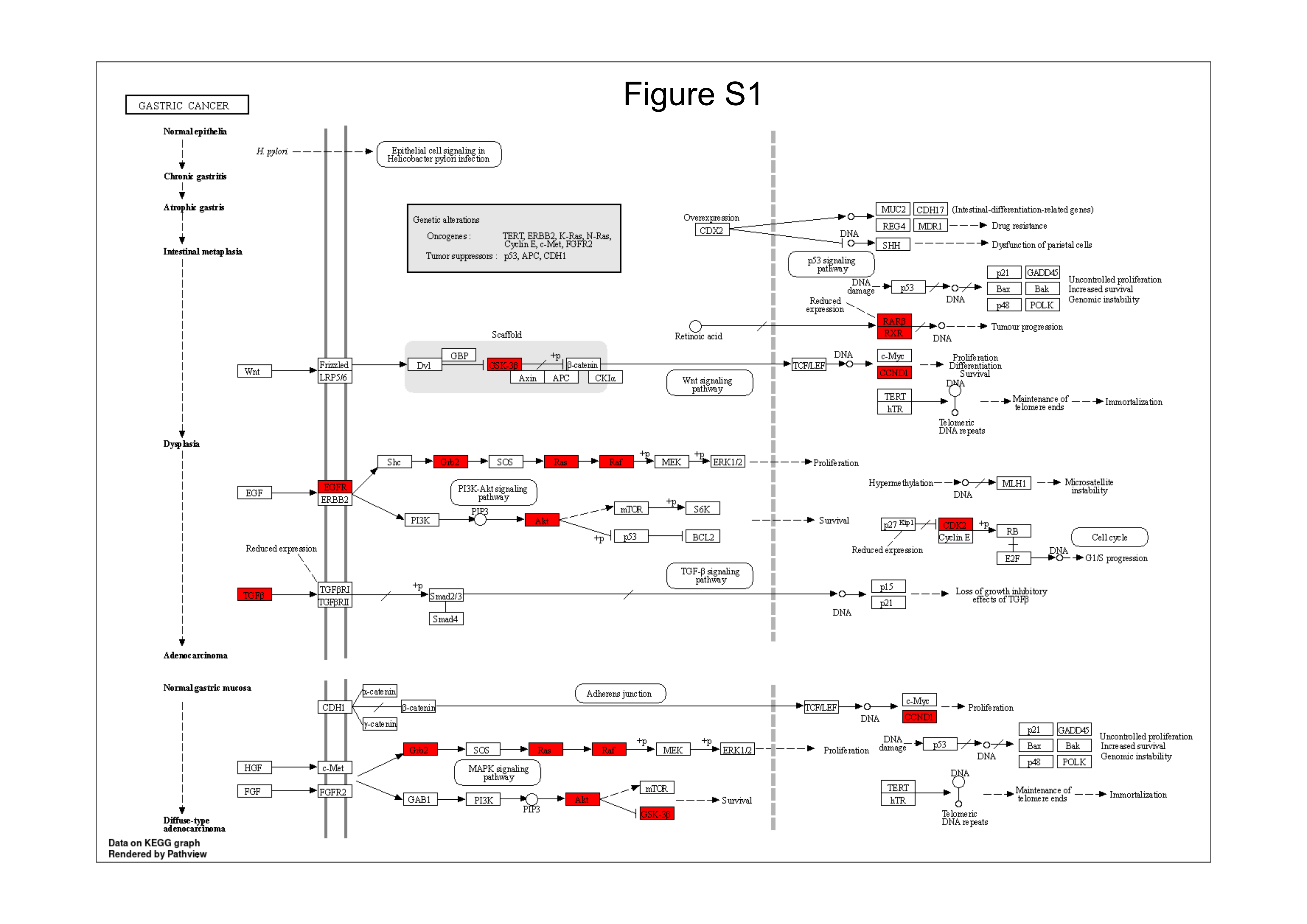

Supplement: Supplementary file 1 — Supplementary Figure S1. [file 41598_2024_62461_MOESM1_ESM.tif]
