# Supplementary material for: Aspartame carcinogenic potential revealed through network toxicology and molecular docking insights
Source: Sci Rep. 2024 May 20;14:11492. doi: 10.1038/s41598-024-62461-w (PMC11106323; doi:10.1038/s41598-024-62461-w)
Supplement: Supplementary file 2 — Supplementary Legends. [file 41598_2024_62461_MOESM2_ESM.docx]

**Supplemental Figure Legends**

**Figure S1. KEGG pathway map of core targets involved in the development and progression of gastric cancer**

This figure represents a signaling pathway illustration of the gastric cancer pathogenesis mechanism. It displays the molecular events and signaling transduction pathways that lead from normal gastric mucosa to the development of gastric cancer, encompassing factors such as genetic mutations, activation or inhibition of signaling pathways, and changes in protein expression. For the content highlighted in red, the figure prominently displays several key molecules or pathways, indicating their states of overexpression or reduced expression, as well as the related effects.
